# Meaning in Life Mediates the Association between Environmental Engagement and Loneliness

**DOI:** 10.3390/ijerph18062897

**Published:** 2021-03-12

**Authors:** Fanli Jia, Kendall Soucie, Kyle Matsuba, Michael W. Pratt

**Affiliations:** 1 Department of Psychology, Seton Hall University, South Orange, NJ 07079, USA; 2 Department of Psychology, University of Windsor, Windsor, ON N9B 3P4, Canada; ksoucie@uwindsor.ca; 3 Department of Psychology, Kwantlen Polytechnic University, Surrey, BC V3S 6R1, Canada; kyle.matsuba@kpu.ca; 4 Department of Psychology, Wilfrid Laurier University, Waterloo, ON N2L 3C5, Canada; mpratt@wlu.ca

**Keywords:** environmental action, well-being, loneliness, meaning in life, care for the future

## Abstract

Although the positive outcomes of human–environment interactions have been established, research examining the motivation between engagement in pro-environmental activities and psychological well-being is limited. In this mixed-methods study, the relationship between pro-environmental engagement, meaning in life, and well-being, including loneliness and depression, were investigated in a sample of 112 young adults in Canada. It was found that engaging in pro-environmental activities was negatively associated with loneliness. This association was mediated by meaning in life (e.g., an intrinsic motive of caring for future generations). In addition, qualitative analyses explored how engaging in pro-environmental activities has a meaningful impact on meaning in life, and on well-being. A thematic analysis generated three unique themes: (1) responsibility to teach the next generation about the environment, (2) deep appreciation for and connection to nature, and (3) renewed agency through self-directed learning. Overall, findings suggest that meaning in life is a core motive that underlies the association between environmental engagement and loneliness. The present study enriched the relationship between pro-environmentalism and well-being with a mixed-methods perspective.

## 1. Introduction

The synergies of human–nature interaction and psychological well-being have gained considerable attention in the fields of Psychology and Public Health. Recent studies indicate that the interaction between humans and nature promotes increased well-being, physical activity, improved quality of life, and may reduce levels of depression and loneliness, e.g., [1,2,3,4,5,6]. However, human–nature interactions exist in many forms, such as experiencing and/or connecting with nature; visiting parks; walking in green spaces; and engaging pro-environmental activities [1,2,3,4,5,6]. There is limited research that empirically examines how protecting the natural environment is related to psychological functioning. Although some pro-environmental activities such as recycling, donating, saving wild species, or organizing an environmental petition do not necessarily involve outdoor engagement with nature, individuals may view these activities as meaningful for themselves and for future generations. In this study, we examined the relationship between engaging in pro-environmental activities and well-being (depression and loneliness). More importantly, we investigated one possible motivational factor, meaning in life, as a potential mechanism underlying this association [7].

Pro-environmental activities can be perceived as meaningful and intrinsic experiences with nature [8,9]. For example, Marshall et al. [10] found that pro-environmental behaviors and climate change beliefs were related to a set of altruistic orientations such as understanding, empathy, and concerns for others. Krettenauer et al. [11] found that participants from Canada and China strongly believed that nature has intrinsic value and that humans have moral obligations to protect the natural environment. In addition, Jia et al. [12] found that environmental activists reported more intrinsic motivations such as self-transcendence, while environmental non-activists reported extrinsic motivations such as self-enhancement. Other studies also suggested that engaging pro-environmental activities should generate meaningful experiences that elicit positive feelings (such as awe, satisfaction, and enjoyment) [3,5,6].

Past studies have also shown that people who engage in more environmentally friendly eco-behaviors experienced heightened well-being in general [1,2,3,4,5,6]. For example, Brown and Kasser [1] found a positive association between pro-environmental behaviors and subjective well-being in a sample of adolescents and adults. Taufik et al. [13] found that, when people are motivated by intrinsic rather than extrinsic rewards, i.e., behaving in ways to preserve the environment, they experience a warm glow, or positive feelings about one’s contribution to the environment. Hartmann et al. [14] also found that a warm glow was derived from personal engagement in pro-environmental behaviors such as climate protection. Similarly, Xiao and Li [15] found that buying environmental products predicted greater life satisfaction, after controlling demographic variables among Chinese consumers.

However, no study has explored a potential mechanism underlying the association between pro-environmental actions and psychological well-being such as loneliness and depression. One possible factor that we propose may help to explain this potential link is meaning in life such as generativity [16]. Generativity is the 7th stage in Erikson’s eight stage life span theory of ego development. It is defined as caring for future generations and leaving a legacy to future as an extension of the self. McAdams [7] expanded on Erikson’s conceptualization by focusing on seven interrelated features, one of which is generative concern. Generative concern is a person’s dispositional tendency to be committed to future generations as meaning in life. In fact, generative concern in relation to environmental issues was first conceptualized as an intrinsic meaning to preserve the natural environment for future generations [17,18]. Several recent studies have found that environmental activists tended to express generativity and cooperativeness as an intrinsic motive to act pro-environmentally in comparison to non-environmental activists [12,19]. Individuals who were experimentally primed to be generative were more likely to donate and donated more of their earnings to an environmental agency as compared to people in the control group [20]. Thus, generative concern is a robust motivation of acting pro-environmentally.

Moreover, researchers have also indicated a positive relationship between generativity and well-being and meaning in life [21]. Ackerman et al. [22] found that generative concern was positively associated with subjective well-being such as satisfaction with life, positive affectivity, and work satisfaction in a sample of young and midlife adults. Similarly, Rothrauff and Cooney [23] found generativity positively predicted a wide range of aspects related to psychological well-being such as self-acceptance, autonomy, personal growth, purpose in life, and positive toward others across adulthood. Further, Lawford and Ramey [24] found that generativity was positively associated with meaning-making within youths’ activity narratives, defined as feeling a sense of purpose while engaging in a positive manner with others and the world in a group of young adults. Thus, it is reasonable to argue that generative concern as meaning in life is one core motive in mediating the association between engaging in pro-environmental activities and positive psychological well-being.

The present study used a mixed-methods approach [25] to investigate the relationship between pro-environmental engagement, meaning in life (generativity), and psychological well-being, including loneliness and depression. We hypothesized that pro-environmental engagement would be negatively related to loneliness and depression, consistent with past research. Second, we investigated whether generative concern served a potential mechanism underlying this association via mediational analyses. Then, we utilized a qualitative approach to iteratively, and inductively capture how participants reflected on their personal, and meaningful lived experiences with nature, and how those experiences extend to future generations through a reflexive thematic analysis (RTA) [25].

## 2. Materials and Methods

### 2.1. Participants

The present study was part of a larger longitudinal project examining the transition to adulthood in Canadian youth (“The Future’s Study”) [26]. In the “Future’s Study”, five rounds of data collection were conducted across a 15-year span. The current data were based on the most recent data collection which both qualitative (narrative) and quantitative data about environmental activities and experiences were collected. One hundred and twelve people completed both questionnaires and interviews about environment and well-being. The sample (*n* = 112) included young adults in Canada (71% women); *M*_age_ = 31.59 years, *SD* = 0.87 years. Of these, 51% of the participants reported that they have at least one child; 83% were full-time employed, 68% had completed a college degree. While the majority participants were European Canadians (91.1%), ten participants identified themselves as members of underrepresented communities (e.g., Black, Chinese, Indian, Hmong, etc.). Portions of these data were analyzed in other studies that had different foci [26].

This study was approved by the Review Ethics Board at Wilfrid Laurier University, Canada. Participants were contacted either by email or phone and invited to participate in Round 5 of the Future’s Study. The study was conducted in-person in participants’ homes or at a private research space on a university campus in Southwestern Ontario, and involved a narrative interview followed by a brief survey instrument that included the following measures.

### 2.2. Questionnaire

Pro-Environmental Activities: Engaging in pro-environmental activities was measured by the Environmental Inventory of Involvement Scale [27]. It consisted of 11 items (e.g., “Contributed time or money to an environmental or wildlife conservation group”, “Taken steps to reduce energy use—turn off water, lights”) that assessed a range of pro-environmental practices. Participants responded on a 4-point Likert scale from 0 (never) to 3 (often) with a Cronbach’s alpha in this sample of 0.83.

Loneliness: Loneliness [28] consisted of 20 items (e.g., “People are around me but not with me”) to assess individuals’ level of loneliness, disconnectedness, and lack of social relations. Participants responded on a 4-point Likert scale from 0 (never) to 3 (often) with a Cronbach’s alpha of 0.92.

Depression: Depression was measured in the Center for Epidemiological Studies Depression Scale (CES-D) [29]. It has 20 items (e.g., “I was bothered by things that usually do not bother me”) to assess self-reported depressive symptoms in a general population. Participants responded on a 4-point Likert scale from 0 (rarely or none of the time; less than 1 day) to 3 (most or all of the time; 5–7 days), with a Cronbach’s alpha of 0.92.

Generative concern: Generative concern was measured with the Loyola Generativity Scale (LGS) [6]. Participants responded to the 20 items LGS using a 9-point scale, ranging from 1 (very strongly disagree) to 9 (very strongly agree). A sample item was “I try to pass along the knowledge I have gained through my experiences.” Responses to items were summed together into a total score. Cronbach’s alpha for this measure was 0.89.

### 2.3. Interview Protocol

As part of a longer interview that captured domains of participants’ life stories [30], participants were asked to recount a significant environmental scene that stands out as meaningful from their lives: “I’d like you to tell me about a time that was meaningful or important in some way with respect to your feelings about environmental issues or the environment”. While participants were encouraged to freely tell their narratives in an unstructured format, the interviewer also asked follow-up and clarifying questions (e.g., can you tell me more about that, what was that like for you, can you elaborate). After telling their narrative in great detail, additional probes such as “please describe what happened, with whom, when, what you were thinking and feeling, the impact the event had on you, and what it says about you as a person” were included in order to glean more information about the experience, and it’s significance or relevance in participants’ lives. Interviews were conducted verbally and participants were compensated for their time with Canadian $50. Interviews were audio-recorded and transcribed verbatim in a Microsoft Word document by a transcriptionist following orthographic transcription guidelines. The first author checked the completed transcripts for accuracy. Each interview was identified by a unique identifier code and all identifying information was removed from the transcripts.

### 2.4. Study Design

We utilized a convergent-parallel mixed method design which involves collecting, analyzing, and integrating both quantitative and qualitative data in a single study [25]. We gave equal priority to both methods and our rationale for mixing was to provide a more complete picture of our findings. As our analytical strategy reflects the integration of hypothesis testing and generation, we first tested associations between pro-environmental activities, generative concern, and well-being in a mediation analysis quantitatively. Then, we analyzed the qualitative narrative interview to support the quantitative findings. We identified common themes to explore how engaging in pro-environmental activities have meaningful impacts on meaning in life, and on well-being.

### 2.5. Data Analyses

Both univariate and multivariate statistical procedures were used to analyze the quantitative data using SPSS version 25. We first computed descriptive statistics and assessed the data for any violations of assumptions of the general linear model (GLM). All dependent variables were normally distributed via visual inspection of the histograms and normal-probability-plots. A z-score cut off criteria of ±3.29 was used to detect outliers. No outliers were present in the dataset, and skewness and kurtosis values were also in acceptable ranges, with values falling between −2 to +2 for skew, and −3 to +3 for kurtosis. No multivariate outliers or influential observations were detected, using a Cook’s distance value of 1.0, and all relationships were linear, were homoscedastic, and normally distributed via inspections of the scatterplots and residual histograms. Descriptive statistics (means, standard deviations, and correlations) and mediation analysis (PROCESS Macro Model 4) [31] were conducted in SPSS version 25.

To further unpack these associations, we identified 20 individuals as a high on environmental engagement (the participants scored over one standard deviation above the mean on pro-environmental engagement). This targeted identification procedure has been used in previous studies [32] to further clarify the quantitative results. Narratives were analyzed using Braun and Clarke’s reflexive thematic analysis framework (RFA) [33]. This approach systematically explores various patterns of meaning, semantic or latent, across participants’ experiences. It allowed us to uncover collective or shared meanings through active engagement with the data, inductively (i.e., from the ground up). While the approach can be atheoretical, we situate our analysis within a social constructionist framework that posits that reality is constructed within the mind of the individual from experiential knowledge, and meaning is made through reflection and narration [34].

With this framework in mind, Braun and Clarke [33] recommended a six-phase, iterative approach to data analysis that involves the following sequence: becoming familiar with the data, generating initial codes, searching for latent meta-themes based on clusters of manifest codes that are conceptually similar, reviewing and revising themes, determining a distinct label for the themes, and then producing a report with exemplar quotes that capture both the breadth and depth of each theme. The first author, who led the thematic analysis, collated the narratives in one Microsoft Word document. He then re-structured the data into a table with three columns: the left column and the right columns were blank, and the narratives were located in the center column. Then, the first author, the second author, and two graduate students, first read and then re-read the narratives in one batch and jotted down initial observations and reactions in a separate Microsoft Word document. They then met as a group to discuss those early thoughts, reactions, and intuitions. They also took notes from this meeting and saved them on the first author’s laboratory computer. Then, they independently coded all of the stories line-by-line, with a focus on the meaning/impact that the environmental activities had on participants’ personal well-being (rather than coding different types of environmental activities), in the left-hand column of the Word document. The group then met again and gained consensus on initial codes, which were then grouped into meaning units based on conceptual similarity in the right-hand column of the word document. Together, they built themes from these conceptual clusters, and rearranged them using a whiteboard. Throughout this process, they continued to document their decisions, and documented reflective notes regarding their decisions, saving all memos and notes on the first author’s laboratory computer. They continued to cross-check their themes regularly against the data.

## 3. Results

### 3.1. Quantitative Results

Descriptive statistics and correlation coefficients can be found in Table 1. Engaging pro-environmental activities was significantly correlated with generative concern, loneliness, and gender (women scored higher). Pro-environmental engagement did not correlate with depression, however. In addition, number of children was negatively correlated with loneliness. Thus, gender and number of children were controlled in further analyses. As we expected, pro-environmental engagement negatively predicted loneliness (*b* = −0.23, *p* = 0.02) after controlling gender and the number of children (*R*^2^ = 0.10, *p* < 0.01). Unexpectedly, there was no significantly direct relationship between pro-environmental engagement and depression (*b* = 0.06, *p* = 0.54). Thus, the mediation analysis was conducted only on the relationship between pro-environmental engagement and loneliness.

To examine whether generative concern mediated the association between pro-environmental activities and loneliness, we used Hayes’s PROCESS Macro v3.1 [31], model 4, which is an observed variable ordinary least squares regression path analysis tool used for estimating direct and indirect effects. Direct effects were tested via 5000 bootstrap samples for bias correction and to establish a 95% confidence interval for the upper and lower limits of the indirect effect. As predicted, generative concern fully mediated the association between pro-environmental engagement and loneliness after controlling for the covariates of gender and number of children (see Table 2). The direct relationship between pro-environmental engagement and loneliness was no longer significant (a full mediation via generative concern). Gender and number of children remained as a significant predictor for loneliness.

### 3.2. Qualitative Results

Using the Braun and Clarke’s [33] qualitative data analytical procedure, three themes were identified: (1) Responsibility to teach the next generation about the environment, (2) deep appreciation for and connection to nature, (3) renewed agency through self-directed learning.

*Responsibility to teach the next generation about the environment.* Several pro-environmental participants expressed the importance of teaching pro-environmental activities to their children, so that these activities will not only carry forward to their children’s lives, but also create a sustainable world for future generations.

“I try to teach my kids about not just the recycling but the composting and the green bin and the importance of how it all fits together and to make the world a better place for them, and when they have kids and their kids.”

(Participant #62) (Participant’s ID code was a random number in the original project.)

These stories connected environmental events and activities (e.g., recycling, conserving energy, climate change) via teaching and learning with their children. These pro-environmental stories increased expectations regarding the quality of the bond and the sense of belonginess with one’s children in later life. These intergenerational responsibilities may promote well-being, as expressed by the following participants:

“I do what I can do so that I can have a better life in this world… my offspring and my offsprings’ offspring, so I do my part.”

(Participant #4)

“I’m committed to do something about climate change, and I hope that I don’t have to apologize too much to my grandkids about it.”

(Participant #458)

“I think more not for myself but sort of looking at my kids and wondering how things will be as they grow up and then their children and sort of more in a long-term scale. Like what can I be doing now to ensure that things aren’t incredibly messed up for them as they get older. So just started to do research into clean energy, climate, like pollution control, looking at alternative energies, things like that.”

(Participant # 186)

*Deep appreciation for and connection to nature.* Another mechanism that connected pro-environmental engagement and well-being, through generative concern, is a connection to nature.

“I enjoy going out there and my brothers and I would ride our bikes out there... part of that is monitoring for areas of high amounts of road kill so that we can provide passage for those creatures so that there isn’t that road kill, that we don’t lose them and that there’s no more frog pavement. So, it was a different time and now people care about the frogs, so it’s part of everything we do now.”

(Participants #633)

“We spent a lot of time on site at xxx in the tailings ponds, which are these big… So we’re spending a lot of time sampling those and it was just the smell combined with just the scene of desolation… if you ever see wildlife that’s struggling, you have to help it. You have to call someone and they have to come and help because sometimes birds will land there. It’s an important migratory route and so they get confused and they land in these ponds and they can get, well, they can die.”

(Participant #672)

“It wasn’t until after I got back from Guatemala, a hurricane hit the community where I had been, and there were mudslides and people died. I had never been, so closely impacted by climate change, because it’s kind of an abstract kind of environmental issue, it was a moment of clarity where I thought that is the environmental issue that I want to work on… the earth is hurting, there’s no lack of environmental tragedies. But it wasn’t until I knew people that had been impacted by it, that I thought, my God, if I can, if I have the luxury to pick what I work on”.

(Participant #833)

“For me, the impact has just been again, to try and appreciate every day what we have, and the fact that for so many people they didn’t, I mean one day they had everything and the next day they had nothing, so just sort of appreciating day to day… everything that I have and, I haven’t had to go through such devastating sort of environmental disaster like that.”

(Participant #765)

These stories illustrated connectedness with nature via outdoor activities in the natural world and personal experiences during natural disasters. Their experiences impacted participants’ views about nature in terms of concern for wild species and gaining a feeling of appreciation.

*Renewed agency through self-directed learning.* Another mechanism between engaging in pro-environmental activities and well-being may be conveyed via learning about the environment. For example, participants described learning activities that were directed toward the natural environment and how it impacted their personal views about nature.

“I am learning a way of sort of promoting new technologies that are gonna help to move us into a carbon-free society.”

(Participant #600)

“I’m very engaged in reading about different environmental issues, politically, socially, green political theory… ecological philosophy and environmentalism and I started to read all this stuff…it’s actually very much integrated into my life and who I am and what I find important and I’ve changed everything from the toiletries I use to the cleaning products, to how I spend my free time to the issues I care about.”

(Participant #648)

“I ended up going back to studying water quality and what I do now as my current job as an educator is to discuss natural resources like the oil sands, talk about their environmental impacts and try and help students understand that maybe there are other ways we can get our energy without having such a big environmental footprint.”

(Participant #672)

“I wrote poetry about animal rights and got in an animal rights book and it was just kind of a pivotal moment because I realized what effect humans had in the bigger picture, not just a human centric world but a complete environmental landscape. And I realized our impact and what we took for granted and really the interplay of the environment on humans’ lives and how we used it and abuse it and what we can change.”

(Participant # 714)

Other participants started with a negative belief such as helplessness and changed their attitude to searching for positive solutions as well as making contributions toward environmental issues.

“The negative sides of it, but here’s all the positive sides of it. And trying to say, okay here’s the problem but here are a few ideas of a solution. Or looking at climate change and saying okay, here’s the problem, but here are some ideas of solutions that could help. They may not fix everything, but help. So that’s more the issue, is his take on the environmental issues was only from the negative…Trying to find the positive things rather than just the negatives… understand the negative but then move forward with it.”

(Participant # 678)

“I was just happy that we were actually cause sometimes you feel like you don’t have a voice and you can’t impact things because the company that produced bottle water have so much more money than the companies that are advertising against bottled water and talking about how ridiculous it is and so I was feeling a little bit of obviously like satisfaction and redemption at being able to make an impact.”

(Participant # 706)

## 4. Discussion

The topic of human-nature connection and aspects of psychological health and well-being have been studied in the field of environmental psychology, e.g., [1,2,3,4,5,6]. However, very few studies have connected engaging in pro-environmental activities and well-being, with a focus on meaning in life (e.g., preserving the environment for the next generation). Pro-environmental engagement can be viewed as an aspect of social engagement that produces meaning and a sense of purpose for individuals. In the current mixed methods study, we explored if generative concern was a motive that fostered well-being in a community sample of Canadian young adults. We found that generativity fully mediated the association between pro-environmental engagement and loneliness. To follow-up these associations, we then searched for possible explanations of these findings by conducting a thematic analysis on a subset of qualitative data, i.e., on a significant environmental scene that participants recalled from their past in a life story interview.

### 4.1. Environmental Engagement, Meaning in Life, and Well-Being

Our findings that pro-environmental activities were directly associated with well-being (e.g., loneliness) support past literature. However, the mechanism underlying this association has yet to be ascertained. Past research has indicated that generative concern is a motive of pro-environmental behavior [17,18,32,35,36]. At the same time, generative concern has been associated with general well-being [37,38,39]. Generative concern may thus serve as a bridge between these two literatures. Indeed, we found that not only did generative concern mediate this association, but participants’ environmental narratives further supported this presumption.

Our thematic analysis revealed three themes that specified the unique impact that participants’ environmental scenes had on meaning in life: (1) teaching pro-environmental activities to children (2) a deep appreciation for and connection to nature, (3) renewed agency through self-directed learning. Even though participants did not directly say their generative concerns from pro-environmental activities reduced their loneliness, they all mentioned that the importance of passing on a sustainable environment for their children and for future generations. This sense of intergenerational responsibility has been found to promote greater life satisfaction, eudaimonic happiness, purpose, autonomy, identity, personal growth, and self-acceptance [23,40]. In addition, pro-environmental individuals reflected on environmental activities as a path to building a personal connection and deep appreciation for preserving natural world. Participants in our study viewed this connection as an opportunity to learn more about nature and make a positive contribution to the environment. These meaningful pro-environmental activities fostered positive feelings and values toward nature which, in turn, was associated with less loneliness. 

The extensive literature on nature and well-being supports this relationship [1,2,3,4,5]. A classic example is the biophilia hypothesis [41]. This hypothesis posits that people’s psychological well-being is associated with their relationship to nature. In addition to simply “feeling good” by experiencing outdoor activities (i.e., experiencing a warm glow), the qualitative analysis demonstrated that these experiences contributed to “living a fulfilled life” [42], i.e., learning from, appreciation of, and contributing to the natural world. Pro-environmental activities thus involved a sense of meaningful connection and subsequent purpose in something larger than oneself, transcending self-interest and fostering compassion and appreciation for the natural world [43].

### 4.2. Strengths of the Mixed-Methods Design

Past research has predominantly used quantitative analysis (such as survey) to establish the relationship between pro-environmentalism and well-being. The present study utilized a convergent-parallel mixed method design to investigate motivations and mechanisms of the relationship. Specifically, we first analyzed the quantitative data to explore possible links among pro-environmental engagement, generative concern, and loneliness/depression. Then, we collected qualitative interviews to support the link and to further explore the process and motivation on how engaging in pro-environmental activities promote well-being via meaning in life. This mixed-methods design can promote a greater understanding of our quantitative analyses and enrich with compressive narratives.

### 4.3. Limitations

While the current study integrated qualitative and quantitative data to unpack these associations, it has several limitations. First, this study was part of a longitudinal project that examined positive development during the transition to adulthood [26]. Measures and data structures were not initially designed to connect pro-environmental engagement and well-being. Therefore, select measures of well-being (depression and loneliness) were used, across each time point. Second, the study only included 112 participants in the mediation analysis. The mediation result might thus be underpowered. Third, although the qualitative interviews helped us to contextualize the mechanism between pro-environmental engagement and well-being, the results were based on a selective sample of participants who endorsed high scores on environmental engagement. A diverse and representative sample is required. In addition, the current study assumed that pro-environmental engagement generated intrinsic motivation (e.g., meaning and purpose in life), which in turn prompted less loneliness. However, this direct hypothesis could not be tested with the current data because the project was not proposed to examine the environment-health outcomes initially. Fourth, we only tested a single construct of generativity (generative concern), on the LGS, and we thus recommend future researchers examine the unique role of action, strivings, and accomplishments, in underpinning these associations [30]. Finally, we asked participants to reflect on significant and meaningful environmental scenes; however, other narrative elicitations could serve useful in expanding on these associations (e.g., early childhood experiences with nature, low-point narratives, etc.).

### 4.4. Future Research

Even though the current study revealed that engaging pro-environmental activities is related to loneliness via meaning in life, future studies need to investigate other aspects of psychological well-being such as life satisfaction, positive emotions, and eduaimonic well-being [23,39,42]. In addition, environmental engagement may also relate to other benefits such as a balanced lifestyle behaviors, cognitive strategies, and physical well-being [43,44,45,46,47]. Future research should also investigate relationship more directly by specifically asking participants about the direct physical and psychological benefits of being engaged in pro-environmental activities either through qualitative or quantitative assessments. Future studies may consider disentangling the role of meaning in life from aspects relating to environmental attitudes, identity, and environmental justice and exploring how these constructs are associated with aspects of psychological well-being and community contributions [48].

## 5. Conclusions

In summary, we demonstrated that generative concern underlies the association between environmental engagement and well-being. Engaging in environmental activities predicted higher levels of generativity which, in turn, protected against loneliness. Qualitative analysis revealed environmental actions led to passages that speak directly to the importance of meaning in life such as intergenerational connection, i.e., imparting lessons to children about nature, a deep respect and appreciation for the natural world that extends beyond the self, and an intrinsic drive to continue to learn more about the environment. Environmental involvement is thus one avenue for the expression of meaning in life in adulthood.

## Figures and Tables

**Table 1:** Descriptive and correlations among variables.

	*M* (*SD*)	1.	2.	3.	4.	5.
1. Loneliness	0.66 (0.50)	-				
2. Depression	0.57 (0.46)	0.52 **	-			
3. Pro-environmental Engagement	1.92 (0.77)	−0.21 **	−0.02	-		
4. Generative Concern	6.76 (1.00)	−0.60 **	−0.26 **	0.38 **	-	
5. Gender	-	0.00	0.23 *	0.20 *	0.26 **	-
6. Number of Children	0.75 (0.84)	−0.21 *	−0.04	−0.01	0.12	0.17

* *p* < 0.05; ** *p* < 0.01.

**Table 2:** Model parameters for mediation analysis on loneliness.

Loneliness	*b* (*SE*)	LLCI	ULCI	*t*	*p*
Constant	--	1.92	2.99	9.19	0.000
Pro-Environmental Engagement	−0.01 (0.05)	−0.11	0.10	−0.17	0.87
Gender	0.19 (0.04)	0.04	0.39	2.46	0.016
Number of Children	−0.17 (0.05)	−0.19	−0.01	−2.28	0.025
Index of Mediator	−0.21 (0.06)	−0.33	−0.10	--	--

Final model *R*^2^ = 0.40, *p* < 0.01.

## Data Availability

The data presented in this study are available on request from the corresponding author. The data are not publicly available due to privacy.
